# Getting Into the Zone: A Pilot Study of Autonomic-Cardiac Modulation and Flow State During Piano Performance

**DOI:** 10.3389/fpsyt.2022.853733

**Published:** 2022-04-13

**Authors:** Shreya Jha, Nicolette Stogios, Adriana Sarmento de Oliveira, Scott Thomas, Robert P. Nolan

**Affiliations:** ^1^Temerty Faculty of Medicine, University of Toronto, Toronto, ON, Canada; ^2^Faculty of Music, University of Toronto, Toronto, ON, Canada; ^3^Institute of Medical Science, University of Toronto, Toronto, ON, Canada; ^4^Heart Institute (InCor), University of São Paulo Medical School, São Paulo, Brazil; ^5^Faculty of Kinesiology and Physical Education, University of Toronto, Toronto, ON, Canada; ^6^Cardiac eHealth and Behavioural Cardiology Research Unit, University Health Network (UHN), Toronto, ON, Canada; ^7^Department of Psychiatry, University of Toronto, Toronto, ON, Canada

**Keywords:** flow state, music performance anxiety, heart rate variability, sympathetic nervous system, autonomic-cardiac modulation

## Abstract

**Background:**

Music performance anxiety is a common experience among elite and professional musicians and impedes performers from achieving flow state, or a state of focused, sustained engagement that promotes optimal performance.

**Objective:**

The aim of this study was to use heart rate variability (HRV) to determine the psychophysiological underpinnings of optimal music performance.

**Methods:**

We assessed HRV to study how autonomic-cardiac modulation was associated with flow during piano performance. Twenty-two pianists (15–22 years) with at least a Grade 8 Royal Conservatory of Music certification prepared two standardized pieces and a self-selected piece. Performer heart rate data were measured with a Polar 800 watch in 5-min periods immediately before performances, during performances and post-performance. HRV was employed to assess autonomic modulation of cardiac intervals. HRV indices of sympathetic and parasympathetic modulation of the heart were analyzed in 2.5-min segments to monitor short-term autonomic adjustments using the Kubios HRV Software. Flow state was measured using the 36-item Flow State Scale (FSS). Relationships were analyzed using zero-order correlations and multiple linear regressions.

**Results:**

Our sample consisted of 22 RCM Grade 8 certified pianists. Participants achieved the highest level of flow during performance of the Bach piece. Decreased HRV was observed during performance, as indicated by a significant drop in total power. Flow state was positively associated with High Frequency (HF) power during the pre-performance phase, and inversely associated with Low Frequency (LF) power during performance.

**Conclusion:**

Inverse association of flow with LF-HRV during performance affirms the importance of vagal-HR modulation for achievement of flow state. Increased HF-HRV and reduced LF-HRV immediately prior to performance suggests that flow state may be shaped as much by physiological preparation during pre-performance as it is by physiologic responses during performance. Further research is required to validate the correlation between autonomic modulation of the heart and flow state. Evidence of this correlation between autonomic modulation of the heart and achievement of flow state may pave the way for further research on enhancing musical performance and targeting MPA through HRV-based interventions.

## Introduction

Performance anxiety is seen in performative tasks such as public speaking and athletics (1). Music performance anxiety (MPA), colloquially known as “stage fright,” is defined as a complex phenomenon involving subjective stress, autonomic arousal, and disrupted behavior upon performing music, usually for an audience, compared to one’s baseline state (2). According to the DSM-5, MPA is considered a subtype of social anxiety disorder (3). MPA is a common concern of professional musicians. In a survey of 650 professional musicians, 60% (*n* = 155) of respondents reported acute symptoms of MPA in anticipation of performances (4). A more recent literature review of 43 studies found that the prevalence of MPA ranged from 16.5 to 60% in diverse musician populations (5). MPA interferes with the performer’s ability to achieve flow state, which is a state of focused, sustained attention and engagement in one’s task (6), combined with the possession of skills to handle the challenges of the task (7). Flow state constitutes an internal psychological and physiological state associated with the ideal of “peak performance” that performers strive for. To date, the psychophysiological responses that facilitate or maintain flow state are poorly understood.

Non-invasive monitoring of autonomic-heart rate modulation *via* heart rate variability (HRV) analysis may improve our understanding of the physiological underpinnings of MPA and flow state. HRV analysis is a reproducible and accessible non-invasive technique that is widely used to assess sympathetic and parasympathetic modulation of the heart (8).

Marked changes in HRV have been connected to anxiety disorders, and particularly social anxiety disorder (SAD). Multiple studies have shown reduced overall HRV and reduced parasympathetic indicators (9–11). Decreased parasympathetic indicators are associated with increased self-reported symptom severity (10). These findings point toward an association of tonically decreased vagal activity with anxiety disorders.

Several studies have explored heart rates during piano performance. Two studies examining professional musicians’ heart rates while performing showed significantly higher heart rates while playing than at rest, as well as significantly higher heart rates during a concert performance than rehearsal (12, 13). Heart rates are also higher while performing higher tempo pieces (14).

Further studies have established a relationship with music and HRV beyond the relationship with heart rate. Firstly, listening to music can affect HRV (15). More specifically, small exploratory studies of HRV during performance have shown a positive relationship between high sympathetic arousal and MPA. A pilot study performed on 11 undergraduate music students showed a shift from high frequency (HF) HRV to low frequency (LF) HRV patterns during high-stress performance, which suggests a shift from parasympathetic to sympathetic modulation of heart rate (16). Similarly, in an observational study that compared rehearsal and competition conditions among 18 pianists, there was increased heart rate and decreased total HRV power during competition, signifying increased sympathetic activity (17).

The physiological manifestation of flow has been assessed with mixed findings. In a study examining flow state in three performance pianists, flow state was positively correlated with HRV markers of sympathetic arousal (18). On the other hand, Harmat et al. observed a pronounced response in HRV indices of parasympathetic activity among three professional pianists during cognitively demanding performances (19). Therefore, while non-invasive monitoring of HRV has provided initial insights into autonomic-cardiac functioning during musical performance, there are limited and conflicting findings regarding the *relationship* between psychological flow and the autonomic-cardiovascular activity in musicians. Further, MPA levels are reported to decrease with pre-performance rituals such as mental rehearsal or meditation (20). This suggests that autonomic activity during the preparatory phase may be as important as during the performance itself in facilitating psychological flow.

The aim of this pilot study was to use non-invasive monitoring of autonomic-cardiac activity and self-reported flow state before, during, and after performance of three separate musical pieces in a group of skilled pianists. We examined the dynamic balance between moderate arousal (reflected by HRV markers of sympathetic modulation and self-reported anxiety levels) and positive valence (reflected by HRV markers of parasympathetic modulation) over the course of performance and their association with self-reported flow state.

## Materials and Methods

### Ethics

This study was approved by the Research Ethics Board of the University of Toronto (protocol number 34638). Participants provided written informed consent.

### Inclusion Criteria

We recruited individuals between the ages of 15–22 who had at least a Royal Conservatory of Music (RCM) Grade 8 piano certification, or the minimum amount required to obtain a high school music credit in Canada and apply to a non-piano undergraduate music program. We excluded individuals who reported an acute or chronic medical condition that affects cardio-respiratory function, smokers, and individuals using prescribed medications known to affect cardiac function. Participants were asked to refrain from alcohol and caffeine for at least 12 h prior to their scheduled session.

### Music Performance Pieces

Participants were asked to prepare Johann Sebastian Bach’s Prelude No. 1 in C Major, Erik Satie’s Gymnopedie No. 1, and a piece of their choice 2 weeks in advance. Bach’s Prelude No. 1 in C Major and Satie’s Gymnopedie No. 1 were chosen due to their clear melodic and harmonic structure and popularity in the world of piano repertoire. Slower pieces were chosen (the Bach piece is usually played at 112 bpm and the Satie at 72 bpm) to avoid the faster heart rate from a faster piece confounding autonomic modulation of the heart brought about by psychophysiological changes. Participants selected a preferred piece as we expected that this performance might yield a higher flow state.

### Participant Baseline Characteristics

The RCM grading system (which runs from Grades 1–10 with two higher certifications after level 10, deemed levels 11 and 12 for the purpose of this study) determined participants’ piano certification. Immediately prior to testing, participants completed the Godin-Shephard leisure time exercise questionnaire (21) and the Hospital Anxiety Depression Survey (HADS) (22). MPA was not explicitly measured as it was not defined as a disease entity. Our focus was the psychophysiological manifestation of flow state rather than that of music performance anxiety.

### Heart Rate Variability

Performer electrocardiogram data was measured with a Polar 800 watch (PolarElectro Oy, Kempele, Finland) for 5 min before performances, during performance and for 5 min immediately post-performance. Its lack of interference with performance made it well-suited to explore MPA. The Polar 800 watch is a non-invasive monitoring device that provides continuous R-R interval data from which autonomic modulation of the heart was assessed. Heart rate variability (HRV) was assessed using Kubios HRV Analysis Software (version 3.0.1, Kuopio, Finland) for 5-min intervals at pre-performance, performance, and post-performance. These intervals were analyzed in 2.5-min segments, in order to detect short-term changes in cardiac-autonomic modulation.

Spectral analysis of the R-R interval segments produced power measures which were interpreted as follows: high frequency peak (HF; 0.15–0.4 Hz) representing primarily parasympathetic modulation, low frequency peak (LF; 0.04–0.15 Hz) reflecting both sympathetic and parasympathetic modulation (23). LF and HF power were each calculated in milliseconds^2^ (ms^2^) and normalized units (nu) (24). The LF/HF ratio was used as a marker of the relative degree of sympathetic-cardiac modulation (24). Total power (milliseconds^2^, ms^2^) was used as an indicator of the overall variability of the RR intervals.

### Flow State

During the final recovery period, performers completed the Flow State Scale (FSS, Supplementary Appendix), a 36-statement questionnaire using a 5 point Likert scale (25). This scale was validated against the State-Trait Anxiety Inventory (STAI) (26) and it has been employed in research with MPA (17, 27).

### Experimental Procedures

Resting HRV was established by monitoring heart rate over a period of 5-min prior to beginning performance. Each participant performed the three above-noted piano pieces on a grand piano in random order, as specified using random.org. Performers were instructed to loop or cut their playing of each piece to provide a 5-min recording. HRV was measured for a 5-min post-performance recovery period and participants completed one FSS questionnaire for each piece. A 1–2-min rest period was subsequently taken between each piece.

### Statistical Analysis

Baseline characteristics and group differences of the sample were evaluated using Pearson’s χ^2^ and one-way analysis of variance (ANOVA) in SPSS 25.0. Data were screened for potential covariates, such as baseline anxiety and depression (HADS questionnaire) (22) and physical activity levels (Godin-Shephard) (21). Mean flow was calculated from FSS total score compared across the three pieces using paired *t*-tests. *Post-hoc* comparisons were applied using the least significant difference due to the small sample and exploratory nature of this study. We compared the percentage of participants experiencing positive flow between pieces using paired *t*-tests. Positive flow was defined as a mean score above 3.5 by creating a mathematical division of the 5-point scale. As a rating of “3” denotes “neither agree nor disagree” with the statement, an average score above 3.5 would signify overall agreement with positive statements about the performance. We examined short-term changes in HRV using mean values for each 2.5-min segment within the 5-min intervals for pre-performance, performance, and recovery.

We selected the piece with the highest level of flow to allow for the most direct examination of physiological conditions that correlate with flow. Pearson correlations were used to identify any potential correlations between flow and HRV indices, as well as baseline characteristics including age, sex, anxiety, depression, and pre-performance HRV components: heart rate (HR), LF and HF power in absolute values (ms^2^), and LF and HF power in normalized units (nu). Normalized HRV measurements were calculated through division of the index (LF or HF) by the short-term frequency bands summated (LF+HF). We examined partial Pearson correlations to determine if HADS scores affected relationships between HRV indices and flow.

Multivariable linear regression was used to examine the independent association between temporal variation in HRV indices across performance intervals and flow. Predictor variables of interest were absolute and normalized LF and HF. We examined whether mean values differed between the pre-performance and subsequent segments, including the latter half of pre-performance and both performance segments.

## Results

Twenty-five participants were enrolled in this study, three of which were excluded from analysis (two due to technical malfunction during data collection and one for extreme HRV values as the result of being an elite athlete). Our final sample size consisted of nine women and thirteen men, ages 15–22 years old (Table 1). There were no significant correlations between age or sex and flow or HRV; therefore, we combined all ages and both sexes into a single group.

**TABLE 1:** Background characteristics of sample.

Sociodemographic features	Mean	SD
Female/male	(13/9)	
Age (Years)	20	1.5
RCM^a^ Qualification (Range: 8–12)	10	1.2
Anxiety: HADS^b^ (Range: 0–21)	9.0	4.2
Depression: HADS (Range: 0–21)	3.3	3.1
Godin Phys total leisure time score (>24 = active, <23 = inactive) (40)	38	28

*^a^RCM: Royal Conservatory of Music.*

*^b^HADS: Hospital Anxiety and Depression Scale.*

Table 2 displays the mean flow of participants during the performance pieces and the percentage of participants exhibiting positive flow. Psychological flow during performance of the Bach piece was significantly higher than that observed in Satie, *t*(21) = -2.51, *p* = 0.02. Furthermore, the prevalence of positive flow (≥3.5) among performers was greater during the Bach performances as compared to the Satie [91 vs. 64%, respectively; *t*(21) = -2.81, *p* = 0.01] while the difference approached significance for the self-selected piece (Own), *t*(21) = -2.02, *p* = 0.057. Given these findings, the Bach piece was chosen for the remainder of analyses of HRV and flow.

**TABLE 2:** Mean flow and percentage of participants experiencing positive flow during performance of the three different music pieces.

Piece	Mean flow	Standard deviation	*p*-value^a^	% of sample experiencing positive flow (>3.5 FSS)	*p*-value^a^
Bach	3.87	0.38		91	
Own	3.80	0.56	0.06	68	0.06
Satie	3.64	0.50	0.02	64	0.01

*^a^P-value denotes paired sample t-test as compared to Bach values.*

Change in HRV indices from the first 2.5-minute pre-performance interval were observed (Table 3). The RR-interval decreased significantly in the second half of pre-performance, *t*(21) = 4.484, *p* < 0.001 and it stayed low throughout performance. Total power decreased significantly from pre-performance 1 to performance onset, *t*(21) = 4.742, *p* < 0.001, while there was no significant change observed between pre-performance 1 to pre-performance 2, *t*(21) = 0.878, *p* = 0.39. LFms^2^ and HFms^2^ also both decreased in line with the drop in total power. Lastly, the LF/HF value decreased due to the proportionally smaller drop in HFms^2^ compared with LFms^2^ over the course of performance.

**TABLE 3:** Selected Heart Rate Variability (HRV) measures during each 2.5-min segment in the pre-performance and performance phases.

Time segment	Pre-performance 1–mean (95% C.I^a^)	Pre-performance 2–mean (95% C.I)	*p*-value^b^	Bach 1–mean (95% CI)	*p*-value^b^	Bach 2–mean (95% CI)	*p*-value^b^
Total power (ms^2^)	3222 (2212, 4233)	2879 (1757, 4000)	0.39	1148 (871, 1425)	<0.001	1647 (1196, 2099)	<0.01
LF (ms^2^)^c^	1558 (827, 2290)	1317 (531, 2103)	0.42	446 (282, 610)	<0.001	605 (351, 859)	<0.01
HF (ms^2^)^d^	515 (325, 705)	364 (244, 485)	0.03	242 (164, 319)	<0.01	253 (154, 353)	<0.01
HF (nu)^e^	26.9 (20.6, 33.2)	30.9 (24.1, 37.8)	0.19	38.1 (30.5, 45.8)	0.02	32.9 (26.4, 39.5)	0.04
RR interval (ms)^e^	722 (681, 763)	695 (658, 733)	<0.01	697 (652, 742)	0.06	697 (657, 736)	<0.05
HR Range (bpm)^f^	65–117	66–114		66–130		69–129	
LF/HF ratio^g^	3.90 (2.35–5.44)	3.25 (1.85–4.6)	0.47	2.68 (1.02–4.3)	0.03	3.27 (1.31–5.22)	0.22

*^a^C.I (confidence interval).*

*^b^P-value denotes paired sample t-test from corresponding pre-performance measurement.*

*^c^LF (ms^2^): low frequency (millisecond squared).*

*^d^HF (ms^2^): high frequency (millisecond squared).*

*^e^nu: normalized units.*

*^f^RR interval: beat-beat interval.*

*^g^HR (bpm): heart rate (beats per minute).*

Correlations were observed among the autonomic indicators at pre-performance, mean flow during the Bach performance, and HADS anxiety score (Table 4). Anxiety was negatively correlated with HFnu and positively correlated with LF/HF ratio. Flow was associated with increased parasympathetic (HFnu) and decreased sympathetic (LFms^2^) influences in the pre-performance condition. Partial correlations controlling for anxiety presented a similar pattern of results (table available upon request).

**TABLE 4:** Zero order correlations for the first 2.5-min segments of pre-performance HRV, Anxiety and Flow during Bach performance.

Variable	Mean flow		Baseline HADS anxiety	
	*r*	Sig (*p* value)	*r*	Sig (*p* value)
**Baseline** HADS^a^ Anxiety	–0.42	0.05	–	–
PP LFms^2^ ^b^	–0.72	<0.0001	0.24	0.29
PP HFms^2^ ^c^	–0.41	0.06	–0.43	0.85
PP HFnu^d^	0.40	0.06	–0.43	<0.05
PP LF/HF^e^	–0.29	0.20	0.44	0.04

*^a^**Baseline** HADS: Baseline Hospital Anxiety/Depression Survey.*

*^b^PP LFms^2^: pre-performance low frequency milliseconds squared.*

*^c^PP HFms^2^: pre-performance high frequency milliseconds squared.*

*^d^PP HFnu: pre-performance high frequency normalized units.*

*^e^PP LF/HF: pre-performance low frequency/high frequency.*

Peak flow was accompanied by decreased LF HRVms^2^ in the pre-performance condition (Table 5), and with increased HF HRVnu (Table 6) prior to performance. Decreased LFms^2^ values prior to performance were associated with self-reported flow state (Table 7). Examination of the second pre-performance condition showed a statistical trend (*p* = 0.1) for a positive association between LFms^2^ activity and flow.

**TABLE 5:** Independent associations of LF/HF ratio with flow during the Bach performance.

LF/HF ratio				
**Variable**	β	**Std. error**	**95% C.I.^a^ (lower, upper)**	**Sig (*p* value)**
(Constant)		0.96	3.90, 4.31	<0.001
Pre-performance1	–0.789	0.042	–0.18, –0.005	0.04
Pre-performance2	–0.173	0.028	–0.08, 0.037	0.43
Bach1	0.547	0.057	–0.060, 0.183	0.30
Bach2	0.166	0.053	–0.096, 0.127	0.77

*Dependent variable = Mean flow of Bach performance.*

*^a^C.I.: confidence interval.*

**TABLE 6:** Independent association of HFnu with flow during the Bach performance in a multivariable linear regression.

HFnu				
**Variable**	β	**Std. error**	**95% C.I.^a^ (lower, upper)**	**Sig (*p* value)**
(Constant)		0.244	3.824, 4.520	<0.001
Pre-performance1	0.843	0.009	0.006, 0.044	0.01
Pre-performance2	–0.277	0.007	–0.021, 0.006	0.28
Bach1	0.022	0.006	–0.011, 0.012	0.93
Bach2	–0.474	0.008	–0.029, 0.004	0.13

*Adjusted R^2^ = 0.164.*

*^a^C.I.: confidence interval.*

**TABLE 7:** Independent association of LF ms^2^ with flow during the Bach performance.

LF ms^2^				
**Variable**	β	**Std. error**	**95% C.I.^a^ (lower, upper)**	**Sig (*p* value)**
(Constant)	4.081	0.099	3.873, 4.289	<0.001
Pre-performance1	–2.58 × 10^–4^	5.7 × 10^–5^	–1.37 × 10^–4^, 0.379 × 10^–4^	<0.001
Pre-performance2	8.24 × 10^–5^	4.7 × 10^–5^	–1.6 × 10^–5^, 1.81 × 10^–4^	0.10
Bach1	2.06 × 10^–4^	2.2 × 10^–4^	–2.62 × 10^–4^, 6.74 × 10^–4^	0.37
Bach2	1.21 × 10^–5^	1.39 × 10^–4^	–2.82 × 10^–4^, 3.06 × 10^–4^	0.93

*Adjusted R^2^ = 0.609.*

*^a^C.I.: confidence interval.*

## Discussion

In this pilot study among elite musicians, we observed that autonomic activity in the pre-performance condition significantly predicted psychological flow during the performance of a pre-selected Bach piece across multiple HRV indices. Peak flow was associated with relatively higher vagal-HR modulation (indicated by HFnu) and lower sympathetic modulation (indicated by LF/HF ratio and LFms^2^). This suggests that increased vagal heart rate modulation and lower sympathetic arousal prior to performance may be necessary to facilitate flow state during performance.

Flow was highest during the Bach piece compared to the self-selected piece and the Satie piece. This is likely primarily due to the popularity of the piece within common piano repertoire, meaning that the majority of participants can satisfy the “challenge-skill balance” dimension of flow (16).

Heart rate variability and RR interval durations decreased significantly from the pre-performance to the performance segment of participant activity. We interpreted this as participants undergoing vagal withdrawal rather than to increased sympathetic drive during this transition, as heart rates increased above 100 bpm but generally remained below 115 bpm (28). The LF/HF ratio was somewhat discrepant with the above pattern, as it decreased from pre-performance to performance. This finding can be attributed to the differential drops in LFms^2^ and HFms^2^ activity; the former decreased by 71% from pre-performance 1 to the beginning of performance while the latter decreased by only 53%.

We observed a significant inverse relationship between flow state and the LF/HF ratio in the pre-performance phase, while observing a trend toward significance for a positive relationship between the LF/HF ratio and flow as well as LFms^2^ and flow during performance. This trend may indicate that sympathetic activity immediately prior to performance and upon onset of performance is beneficial to facilitate flow. As mentioned previously, Manzano et al. (18) found a positive relationship between the LF/HF ratio and flow during performance. However, it is important to note they measured HRV during the performance condition only. As such, the discrepancy between our study and Manzano’s et al.’s study could be attributed to the distinction between pre-performance and performance conditions as well as the difference in demographic features that distinguish professional, adult concert pianists from young adult music students.

Our findings are consistent with common verbal descriptions of psychological flow. The idea of getting “in the zone” during pre-performance [i.e., a state of confidence and relaxation (29)] matches the negative association of flow with LFms^2^ and LF/HF, and its positive association with HFnu in the first half of the pre-performance condition. As the time of performance draws closer, seasoned performers “gear up” their senses to perform and become more physiologically aroused and psychologically alert (30). This state of heightened arousal matches the positive association of LFms^2^ with flow in the second half of pre-performance.

Importantly, the results of our study suggest there may be preventative potential for interventions to exploit autonomic modulation of the heart in order to train performers to facilitate flow state. HRV is a malleable measure that is also affected by several modulators, including overt behavior and cognitive-affective state (15, 31). As the sympathetic nervous system activity has been broadly connected to anxiety responses (9–11, 32), our findings may be particularly useful for those who suffer from music performance anxiety. Acute physiologic arousal associated with stress and anxiety can be mitigated using relaxation or biofeedback-assisted relaxation under conditions that mimic the performance experience (31). One can learn to increase vagal modulation of the heart to counter the effects of stress (33), which suggests it may be possible to use specific targets of vagal-heart rate modulation during pre-performance or performance to improve one’s achievement of flow state. Possible procedures include graduated exposure to stressors or the use of a stress-reactivity paradigm to promote a controlled response to the stress of performance, allowing participants to achieve a focused, calm state under performance conditions (31).

Additionally, our participants were typical of young elite musicians with symptoms of anxiety and depression in line with normative HADS values for students or individuals in the early stages of their career (34). This indicates that the results of this study are likely generalizable to early-career musicians. However, further research is needed to assess the application of our findings to non-elite musicians or seasoned professionals.

Overall, despite the small sample size, our HRV measures during the pre-performance phase showed a significant and independent association with the flow state achieved by participants. However, these measures require replication with a larger sample size to observe the robustness of the association. It is important to acknowledge that the interpretation of HRV has been disputed. While some studies validate its use as an indicator of stress (23) and consider the low and high frequency spectral bandwidths to reflect sympathetic and parasympathetic modulation, respectively (35), others have questioned the ability of the LF bandwidth to reflect sympathetic activity (36). Nevertheless, HRV has been utilized as a clinically relevant tool that can link behavioral modifications to the ANS (31). Moreover, as we did not take a detailed pharmacological history of each participant, one cannot rule out the potential impact of pharmacotherapy on the results. Lastly, the FSS was filled out retrospectively in this trial, which may have impacted the results and would be an important amendment for future trials.

One important future direction concerns the ecological validity of the study. Participants performed in a school classroom with little to no audience (up to three other people, including the experimenters). The lack of a live audience removes one of the key components of a performance that may trigger anxiety. Future studies could make use of a non-invasive measurement apparatus to observe the association between HRV and flow during live performances, such as the study undertaken by Iñesta et al. (13). The setup is easily transferable to a stage and interferes minimally with performance, making it an ideal measurement tool. Future studies may examine the relationship between brain development and flow state, particularly in the context of young adults and teenagers. Lastly, it would be appropriate to repeat this trial with use of questionnaires tailored to music performance anxiety, such as the Kenny-Music Performance Anxiety Inventory (K-MPAI) (37). This would allow for focus and stratification of changes in individuals with high levels of performance anxiety.

Future research could extend to other possible physiological indicators/modulators of flow state, including blood pressure variability or skeletal muscle activity such as frontalis muscle or temporomandibular muscle activity. For example, Manzano et al. (18) found that flow correlated with increased activation of the major facial muscles needed for smiling when examining flow in three performance pianists. Similarly, Kivikangas’s study (38) found that flow negatively correlated with EMG activity in the muscles used in facial expression. Such investigations would allow for multifaceted observations that would help to distinguish physiologic responses that are essential to facilitating psychological flow during performance. Furthermore, use of EEG could provide insights into the neurological correlates of flow state or MPA. Katahira et al. reported that increased frontal theta and frontocentral alpha activity, assessed using EEG, may characterize flow state (39).

## Conclusion

Our pilot study of monitoring of autonomic-cardiac modulation demonstrated sequential shifts in HRV indices that were associated with the achievement of an increased flow state during musical performance, and specifically in the pre-performance or preparatory period. Our results suggest that a global shift toward vagal modulation and relatively lower sympathetic activity during pre-performance may be a necessary condition to facilitate peak flow. The present results contribute to an improved understanding of psychological flow during performance, as well as possible mechanisms to decrease the impact of MPA. Further research is warranted to explore HRV-based therapies for musicians who are required to perform optimally in recurrent stress-evoking situations such as the performance stage.

## Data Availability Statement

The raw data supporting the conclusions of this article will be made available by the authors, upon request, without undue reservation.

## Ethics Statement

The studies involving human participants were reviewed and approved by Health Sciences Research Ethics Board, Faculty of Kinesiology and Physical Education, University of Toronto. Written informed consent to participate in this study was provided by participants.

## Author Contributions

All authors contributed to drafting and revising the manuscript and approved its final version. SJ, RN, ST, and AO were involved in the concept and design of the study. SJ and AO were involved in data collection. SJ, AO, and NS were involved in the systematic search, screening of articles, extraction of data, and the statistical analysis. RN, ST, AO, and NS revised the work critically and provided suggestions for improvement.

## Conflict of Interest

The authors declare that the research was conducted in the absence of any commercial or financial relationships that could be construed as a potential conflict of interest.

## Publisher’s Note

All claims expressed in this article are solely those of the authors and do not necessarily represent those of their affiliated organizations, or those of the publisher, the editors and the reviewers. Any product that may be evaluated in this article, or claim that may be made by its manufacturer, is not guaranteed or endorsed by the publisher.
