# Supplementary material for: Getting Into the Zone: A Pilot Study of Autonomic-Cardiac Modulation and Flow State During Piano Performance
Source: Front Psychiatry. 2022 Apr 13;13:853733. doi: 10.3389/fpsyt.2022.853733 (PMC9044034; doi:10.3389/fpsyt.2022.853733)
Supplement: Supplementary file 1 [file Image_1.PDF]

Appendix

Flow State Scale

Please answer the following questions in relation to your experience in the event you have just completed. These questions relate to the thoughts and feelings you may have experienced during the event. There are no right or wrong answers. Think about how you felt during the event and answer the questions using the rating scale below. Circle the number that best matches your experience from the options to the right of each question.

Rating Scale:

Strongly disagree	Disagree	Neither agree nor disagree	Agree	Strongly agree
1	2	3	4	5

	Strongly disagree				Strongly agree
1. I was challenged, but I believed my skills would allow me to meet the challenge.	1	2	3	4	5
2. I made the correct movements without thinking about trying to do so.	1	2	3	4	5
3. I knew clearly what I wanted to do.	1	2	3	4	5
4. It was really clear to me that I was doing well.	1	2	3	4	5
5. My attention was focused entirely on what I was doing.	1	2	3	4	5
6. I felt in total control of what I was doing.	1	2	3	4	5
7. I was not concerned with what others may have been thinking of me.	1	2	3	4	5
8. Time seemed to alter (either slowed down or speeded up).	1	2	3	4	5
9. I really enjoyed the experience.	1	2	3	4	5
10. My abilities matched the high challenge of the situation.	1	2	3	4	5
11. Things just seemed to be happening automatically.	1	2	3	4	5
12. I had a strong sense of what I wanted to do.	1	2	3	4	5
13. I was aware of how well I was performing.	1	2	3	4	5
14. It was no effort to keep my mind on what was happening.	1	2	3	4	5
15. I felt like I could control what I was doing.	1	2	3	4	5
16. I was not worried about my performance during the event.	1	2	3	4	5

17. The way time passed seemed to be different from normal.	1	2	3	4	5
18. I loved the feeling of that performance and want to capture it again.	1	2	3	4	5
19. I felt I was competent enough to meet the high demands of the situation.	1	2	3	4	5
20. I performed automatically.	1	2	3	4	5
21. I knew what I wanted to achieve.	1	2	3	4	5
22. I had a good idea while I was performing about how well I was doing.	1	2	3	4	5
23. I had total concentration.	1	2	3	4	5
24. I had a feeling of total control.	1	2	3	4	5
25. I was not concerned with how I was presenting myself.	1	2	3	4	5
26. It felt like time stopped while I was performing.	1	2	3	4	5
27. The experience left me feeling great.	1	2	3	4	5
28. The challenge and my skills were at an equally high level.	1	2	3	4	5
29. I did things spontaneously and automatically without having to think.	1	2	3	4	5
30. My goals were clearly defined.	1	2	3	4	5
31. I could tell by the way I was performing how well I was doing.	1	2	3	4	5
32. I was completely focused on the task at hand.	1	2	3	4	5
33. I felt in total control of my body.	1	2	3	4	5
34. I was not worried about what others may have been thinking of me.	1	2	3	4	5
35. At times, it almost seemed like things were happening in slow motion.	1	2	3	4	5
36. I found the experience extremely rewarding.	1	2	3	4	5
